# Gut Microbiota Shifting in Irritable Bowel Syndrome: The Mysterious Role of *Blastocystis* sp.

**DOI:** 10.3389/fmed.2022.890127

**Published:** 2022-06-20

**Authors:** Alireza Olyaiee, Amir Sadeghi, Abbas Yadegar, Elnaz Sadat Mirsamadi, Hamed Mirjalali

**Affiliations:** ^1^Department of Biology, Science and Research Branch, Islamic Azad University, Tehran, Iran; ^2^Gastroenterology and Liver Diseases Research Center, Research Institute for Gastroenterology and Liver Diseases, Shahid Beheshti University of Medical Sciences, Tehran, Iran; ^3^Foodborne and Waterborne Diseases Research Center, Research Institute for Gastroenterology and Liver Diseases, Shahid Beheshti University of Medical Sciences, Tehran, Iran; ^4^Department of Microbiology, Faculty of Medicine, Tehran Medical Sciences, Islamic Azad University, Tehran, Iran

**Keywords:** irritable bowel syndrome, gut microbiota, *Blastocystis* sp., dysbiosis, post-infectious-IBS

## Abstract

Irritable bowel syndrome (IBS) is a chronic disorder, which its causative agent is not completely clear; however, the interaction between microorganisms and gastrointestinal (GI) epithelial cells plays a critical role in the development of IBS and presenting symptoms. During recent decades, many studies have highlighted the high prevalence of *Blastocystis* sp. in patients with IBS and suggested a probable role for this protist in this disease. Recent studies have documented changes in the gut microbiota composition in patients with IBS regarding the presence of *Blastocystis* sp., but it is not clear that either disturbance of the gut during GI disorders is a favorable condition for *Blastocystis* sp. colonization or the presence of this protist may lead to alteration in the gut microbiota in IBS patients. In this review, we comprehensively gather and discuss scientific findings covering the role of *Blastocystis* sp. in IBS *via* gut microbiota shifting.

## Introduction

Irritable bowel syndrome (IBS) is a chronic disorder, which is known by abdominal pain and abnormal defection. IBS seems to be a gut-brain axis-related disease; therefore, it is also called a functional gastrointestinal (GI) disorder (1, 2). IBS is a commonly reported disorder in clinical practices, affecting 10–20% of the world's population (3).

IBS could be asymptomatic; however, this disease is characterized by symptoms such as abdominal pain, variable bowel habits, and bloating (1). IBS is a multifactorial disorder. Even though its causative agent is not clear; gut microbiota disturbance appears to play an important role in this disorder (4). Early studies have documented the role of microbial gastroenteritis [post-infectious IBS (PI-IBS)] (5, 6) and overuse of broad-spectrum antibiotics in the development of IBS (7). Studies on the gut microbiota in patients with IBS suggest that microbial dysbiosis may increase the severity and duration of IBS symptoms. Accordingly, it was documented that changes in the gut microbiota composition play a critical role in establishing, developing, and flaring the symptoms of IBS (8). Nevertheless, the manipulation of the intestinal microbiota can be considered a new treatment method for patients with IBS. For example, fecal microbiota transplantation (FMT) reduced inflammation and symptoms in people with IBS and could be regarded as a treatment strategy (9).

In addition to the critical role of bacteria in the development of IBS, the high prevalence of some protozoa, such as *Blastocystis* sp., has highlighted the putative role of this protist in the development of IBS. Although a bilateral correlation between the presence of *Blastocystis* sp. and the gut microbiota composition has been reported in many studies, it is not clear whether the gut conditions and gut microbiota perturbation lead to higher colonization of *Blastocystis* sp. in the gut or colonization of *Blastocystis* sp. may lead to dysbiosis (10, 11).

## Parasites and IBS

It has been suggested that microbial infections may lead to a mild inflammation through the intestine and the development of IBS. Although the results contradict (12), the emerging role of intestinal parasites in the development of IBS is now being investigated (13). Intestinal parasites can increase the gut permeability and contact of lumen antigens with the lower layers of the intestine, provoking immune responses, and developing chronic inflammation (14, 15).

Accordingly, a higher prevalence of some protozoa (*Giardia, Blastocystis*, and *Cryptosporidium*) was recorded among the patients with IBS compared to healthy controls (16). The correlation between previous infection with *Giardia* and an increased risk of IBS was strongly suggested by retrospective studies. In this regard, in a retrospective cohort study performed by Dormond et al. (17), which was carried out on military personnel, the risk of developing chronic GI disorders, such as IBS, was assessed in those with documented giardiasis and the findings represented an increased risk of IBS in *Giardia*-infected personnel (17). Nakao et al. (18), in a large retrospective study, which employed the 2006–2010 MarketScan commercial insurance database, showed that despite considering confounding factors, such as anxiety, depression, and healthcare utilization, giardiasis increased the risk of subsequent IBS (18). Although the mechanism beyond the role of giardiasis in increasing the risk of IBS is not clear, destruction of the intestinal barrier unity during colonization of *G. lamblia* appears to be an important factor. For example, it was demonstrated that infection by *Giardia* may lead to gut barrier dysfunctions *via* downregulation of tight junctions, such as claudin-1, and induction of apoptosis in epithelial cells throughout the intestine (19).

There is evidence speculating the role of early-age infection by *Cryptosporidium* in the development of IBS in adolescence, *via* increased jejunal sensitivity to bloating (20). In addition, the intestinal stage of a nematode worm, *Trichinella spiralis*, seems to provoke a mild inflammation over the gut epithelial cell, which can lead to visceral sensitivity and changes in intestinal motility (21–23). It was shown that transient mucosal inflammation in Swiss mice during the acute phase of infection by *T. spiralis* can lead to an alteration in neuromuscular functions, even after the resolution of *Trichinella*-caused inflammation (21). These findings were then supported by Venkova et al. (23), who demonstrated jejunal inflammation due to *T. spiralis*, which induced long-term changes in muscle contractility and enteric neurotransmission, even after recovery from mucosal inflammation (23). Eventually, it was demonstrated that during the GI phase of trichinosis, *T. spiralis* can induce a long-term remodeling of epithelial functions (22). Therefore, the persistent inflammation and neuromuscular dysfunctions during the intestinal stage of trichinosis, seem to increase the risk of IBS development, particularly in those who are susceptible.

*Dientamoeba fragilis* is an intestinal protozoan, which, together with *Blastocystis* sp., are the most prevalent protozoa reported in patients with IBS (24–26). The probable correlation between *D. fragilis* and IBS was first reported by Borody et al. (27) who showed that eradication of the protozoan led to amelioration of IBS-like symptoms. However, further investigations on the correlation between *D. fragilis* and IBS have reported controversial results. For example, Yakoob et al. (28) demonstrated a higher prevalence of *D. fragilis* in patients with IBS compared to healthy controls. Engsbro et al. (25) reported that 35–41% of patients with IBS carried out *D. fragilis* in the Danish population, and Ibrahim et al. (29) supported previous studies and documented a higher prevalence of *D. fragilis* in patients with IBS compared to control subjects. A case of PI-IBS due to *D. fragilis* in a patient who traveled to Mexico points out the probable role of this protozoan in the development of IBS, as well (30). However, in contrast to these studies, Engsbro et al. (31) analyzed the response to anti-*D. fragilis* treatment in 25 patients with IBS who carried the protozoan and showed the lack of correlation between microbiological response to treatment and clinical manifestations of patients with IBS.

## The High Prevalence of *Blastocystis* sp. in Patients With IBS

*Blastocystis* sp. is frequently reported from patients with IBS, and numerous studies have suggested a correlation between carrying this protist and IBS; however, it is not clear whether either *Blastocystis* sp. leads to IBS/IBS-like symptoms or perturbed conditions of the GI tract provide a favorable niche for colonization of *Blastocystis* sp. One of the first studies that examined a probable parasite as a causative agent of IBS was performed by Yakoob et al. (32), who reported a statistically significant higher prevalence of *Blastocystis* sp. in patients with IBS [46% (44 of 95)] compared to the controls [7% (4 of 55)]. Surangsrirat et al. (33), in a case–control study in Thailand, documented a higher frequency of *Blastocystis* sp. in patients with IBS (16.7%) compared to the control group (10%), although this difference was not statistically significant. In addition, Das et al. (34) demonstrated that the prevalence of *Blastocystis* sp. was three times higher than that reported in healthy subjects.

The close frequency rate of *Blastocystis* sp. in patients with IBS and control groups in many studies and a greater prevalence rate of *Blastocystis* sp. in control groups compared to patients with IBS have obscured an established linkage between the presence of this protist and the development of IBS (35–37).

The presence of correlation between genetic lineages of *Blastocystis* sp. and the development of IBS has also been evaluated. Although a correlation between the presence of ST1 (38) and ST3 (39) with IBS was suggested, most of the studies failed to associate the presence of a certain subtype with IBS development. In this regard, Pena et al. (40) analyzed the subtype distribution of *Blastocystis* sp. in patients with IBS compared to healthy controls that the results showed the presence of ST1 and ST2 in both groups, while ST3 and ST4 were only characterized by healthy controls and patients with IBS, respectively. The lack of linkage between certain subtypes and IBS was observed in Indian subjects, where ST3 was the dominant subtype in both IBS and control groups followed by ST1 (34). Subtypes 1 and 3 were also reported to be the major genetic lineages in IBS and control subjects in other studies (41–43).

The expression of certain enzymes in *Blastocystis* sp. isolated from patients with IBS was also evaluated. In this regard, Nagel et al. (44) indicated the higher presence of a *Blastocystis* sp. protein (probably a cysteine protease) in subjects with IBS compared to healthy controls. They also claimed that some single nucleotide polymorphisms (SNPs) in IL-8 and IL-10 probably affect the relative risk of IBS development in individuals who carry *Blastocystis* sp. (45). Although the effects of genetic polymorphisms through the IBS-related signature regions of IL-6, IL-8, and IL-10 might be different in various ethnic groups, these SNPs could increase the risk of IBS development (46, 47). In this line, a recently published study proposed significantly higher serum levels of IL-6, IL-8, IL-10, IFN-γ, and TNF-α in patients with IBS who colonized with *Blastocystis* sp., proposing the critical effects of *Blastocystis* sp., in IBS development (48) *via* modulation of IBS-related cytokines (49).

Taken together, although colonization of a certain subtype of *Blastocystis* sp. does not seem to be correlated with IBS, it was suggested that there are significant differences in the protease activity of different subtypes of *Blastocystis* sp. (50). Importantly, proteases released by *Blastocystis* sp. can disrupt the epithelial barrier and actin filaments, increase gut permeability, and subsequently develop IBS (51–53). Moreover, a most recently published study suggested that *Blastocystis* sp. ST3 can modulate the expression levels of microRNAs involved in the gut barrier integrity, and claudin-7 (54). Notably, claudin-7 is categorized among pre-sealing tight junction proteins and plays a critical role in reducing the permeability of the gut (55).

## A Brief Look at the Gut Microbiota

The microbiota is comprised of the microbial community of the human body, which is made up of a variety of microorganisms, including bacteria, viruses, fungi, and protozoa. The gut microbiota consists of between 10 and 100 trillion cells, which are ~10 times the total cells of a human (56). The gut microbiome has the highest number of microbial communities. The number of microbial genes in the gut is estimated to be 150 times higher than the genes of human origin (57). Current documentations demonstrated four bacterial phyla, including Firmicutes, Bacteroidetes, Proteobacteria, and Actinobacteria, are the core of the gut microbiota of which Firmicutes and Bacteroidetes are predominant (58–60).

The gut microbiota composition is linked to a couple of intestinal and extra-intestinal disorders (61–64). Changes in the GI microbiota (is known as dysbiosis) can affect the immune responses, metabolism, and intestinal permeability, resulting in a pre-inflammatory state. Such changes can disrupt the functions of the host's immunity and metabolic systems, which may lead to diseases, such as diabetes, obesity, GI, neurological, and autoimmune disorders (61, 63, 65, 66). A number of studies have demonstrated a link between changes in the gut microbiota and incidences of gut-related diseases, such as obesity, non-alcoholic steatohepatitis (NASH), IBS, inflammatory bowel disease (IBD), celiac disease, and GI neoplasms (61, 66–68).

## The Gut Microbiota in IBS

The correlation between gut microbiota dysbiosis and IBS conditions is well-established (69, 70). A correlation between the severity of IBS and a gut microbiota signature has been demonstrated (71). PI-IBS, small intestine bacterial overgrowth (SIBO), stress, antibiotics, diet, and early childhood experiences shape the gut microbiota and affect the incidence rate of IBS (72).

A gut microbiota analysis suggested a doubled ratio of Firmicutes to Bacteroidetes, an increased number of *Dorea, Ruminococcus*, and *Clostridium* spp., and a decreased number of *Faecalibacterium* spp. in patients with IBS compared to healthy controls (69). Tap et al. (71) reported an association between the gut microbiota composition and the severity of IBS. They showed that enterotypes *Prevotella* and *Bacteroides* represented the lowest and highest proportion in patients with severe IBS, respectively. In addition, it was claimed that the highest and lowest proportion of *Bacteroides* were observed in IBS-D and IBS-C, respectively (71). These results were then confirmed by Zhuang et al. (73) who documented the Bacteroidetes in stool samples of patients with IBS-D, and suggested an association between the gut microbiota composition with the pathogenicity of IBS (73). IBS has four types, which are characterized based on the stool formation and the number of defecation, including IBS-C with constipation; IBS-D with diarrhea; IBS-M, which has intermittent bowel pattern with a mix of diarrhea and constipation; and IBS-U, which is not easily classified into any of the mentioned groups (74). Metagenomics studies suggest that the gut microbiota may present different patterns according to the types of IBS. The results of a quantitative real-time PCR, which was employed to amplify the 16S rRNA gene of the gut bacteria, showed that the number of bacteria, such as *R. productus, C. coccoides, Villonella, Tetiotamicron, Pseudomonas aeruginosa*, and Gram-negative bacteria in patients with IBS was higher, but the number of *Lactobacillus* was lower than healthy individuals. In addition, the number of *Violinella* species in patients with IBS-C and *P. aeruginosa* in people with IBS-C and IBS-D were higher than in healthy individuals (75).

The 16S rRNA gene sequencing revealed that although the gut microbiota diversity was similar between patients with IBS and healthy controls, the richness of bacteria in patients with IBS-D was lower than in other groups, while a significant increase in Proteobacteria and decrease in Firmicutes, Fusobacteria, and Actinobacteria were observed in patients with IBS-D (76). Apart from the changes in the diversity and richness of the gut microbiota, the overgrowth of the bacterial community throughout the small intestine is thought to be associated with IBS (4). A systematic review by Pittayanon et al. (77) on 24 studies revealed that Enterobacteriaceae (phylum Proteobacteria), family Lactobacillaceae, and genus *Bacteroides* were increased, and uncultured Clostridiales I, genus *Faecalibacterium* (e.g., *F. prausnitzii*), and genus *Bifidobacterium* were decreased in patients with IBS compared to healthy controls. Nevertheless, the presence of a microbiome signature, how IBS alters the gut microbiota compositions, and the role of diet changes on the gut microbiota alterations in patients with IBS are the main unclear issues that need to be investigated (Table 1).

**Table 1:** The gut microbiota changes in patients with IBS.

**No**.	**Sample type**	**Age (average)**	**Method**	**Number of patients**	**Number of healthy**	**IBS patients**		**References**
						**Increase**	**Decrease**	
1	Stool	NP	qPCR	47	30	*Lactobacillus* *Ruminococcus* *Veillonella* *Bacteroides* *Pseudomonas* *Clostridium*	*Bifidobacterium Entrococcus*	(75)
2	Stool	Adult (28–59)	16S rRNA gene sequencing	44	47	Bacteroidetes	Proteobacteria Firmicutes	(78)
3	Stool	Adult (22–66)	qPCR, Microarray Analysis	62	46	*Streptococcus* *Clostridium* *Papillibacter* *Ruminococcus* *Sporobacter* *Dialister* *Peptococcus* *Blautia* *Butyrivibrio* *Dorea* *Roseburia* *Lachnospira*	Bifidobacteria *Alistipes* Bacteroidetes *Odoribacter* Parabacteroides *Prevotella* *Faecalibacterium*	(69)
4	Duodenal mucosa and lumen, Rectal mucosa and lumen	Adult	16S rRNA gene sequencing	74	20	*Bacteroides* *Prevotella* *Oscillospira*	–	(79)
5	stool	Adult (37–60)	16S rRNA gene sequencing	20	18	*Enterobacter* *Streptococcus* *Fusobacterium* *Rothia*	*Roseburia* *Faecalibacterium*	(80)
6	Stool, Mucosal Samples	Adults (18–65)	16S rRNA gene sequencing, qPCR	110	39	Bacteroidetes Clostridiales *Lachnospira* *Ruminococcus*	*Faecalibacterium* *Prevotella* Firmicutes *Blautia* *Coprococcus*	(71)
7	Stool	Adult	16S rRNA gene sequencing	27	13	Bacteroidetes Fusobacteria Faecalibacter	Firmicutes Proteobacteria *Lachnospira* *Ruminococcus* *Lactobacillus*	(73)
8	Stool	Adult	16S rRNA gene sequencing	30	30	Proteobacteria Enterobacter Bacteroidetes *Streptococcus* *Lactobacillus* *Escherichia coli* *Proteobacteria* *Enterobacter*	Firmicutes Fusobacteria Actinobacteria *Clostridia* *Ruminococcus* *Faecalibacterium* *B. pseudocatenulatum*	(76)
9	Stool	Adult	Metagenomics gene-targeted approach	3	8	*Dialister* *Faecalibacterium* Alcaligenaceae	–	(81)
10	Stool	Adult (27–46)	16S rRNA gene sequencing	19	16	*Ruminococcus*	–	(82)
11	Stool	Adult/child	16S rRNA gene sequencing, qPCR	74	21	*Escherichia/Shigella* *Aeromonas*	*Actinobacteria* *Citrobacter* *Microvirgula*	(83)
12	Stool, Sigmoid biopsy	Adult/child	454 pyrosequencing	40	20	Bacteroidetes *Prevotella*	Lachnospiraceae incertae sedis, *Coprococcus*,	(84)
							*Clostridium* XI, *Odoribacter*, *Butyricimonas*, *Alistipes*, *Blautia*	
13	Stool	Adult	16S rRNA gene sequencing	16	21	Clostridiales Acidimicrobidae Lachnospiraceae *Clostridiales*	Actinobacteria Rhodospirillales Burkholderiales Cyanobacteria Sphingomonadaceae	(85)

## The Gut Microbiota in IBS and Immune System

There is growing evidence linking inflammation with IBS. Inflammation may lead to changes in smooth muscles and GI nerves, which are resulted in GI dysfunctions (86, 87). The presence and the number of mast cells, release of histamine and tryptase, distance of intestinal nerve to mast cells in patients with IBS characterized by Room II, and abdominal pain were compared to healthy controls that the infiltration of mast cells and release of their contents in proximity to mucosal innervation were probably correlated with IBS and abdominal pain (88).

The correlation between low level of inflammation and IBS manifestations was suggested in a study by Ohman et al. (89) who showed an increased frequency of blood T cells expressing CD69 and integrin b7/HLA-DR. They concluded that T-cell activation supports low-grade inflammation and symptom generation in patients with IBS (89). This finding was later supported by Nasser et al. (90), who showed an immune activation of CD4+ T cell derived from patients with IBS-D, while it was not correlated with physiological stress. However, they suggested that immune activation is could be a trigger of or a parallel phenomenon with IBS (90). The higher number and the activation of mucosal B lymphocytes and plasma cells, together with an increased number of mast cells in the mucosal jejunal biopsy of patients with IBS-D, probably contribute to the presentation of the disorder (91). Therefore, it seems that a mild inflammation due to enhanced humoral and innate immunity throughout the intestine could be correlated with IBS (91, 92). Importantly, amelioration of the clinical manifestations of IBS-D *via* activation of mast cells and modulating of the intestinal innate immunity followed by oral prescription of disodium cromoglycate (DSCG) confirmed the hypothesis of correlation between mild inflammation and IBS development (93).

The role of gut microbiota in the arrangement of the immune responses in IBS is still investigating; however, it seems that microbe-association pattern recognition plays a determinative role in orchestrating the immune responses (94). In this regard, the role of toll-like receptors (TLRs) as cross-road between gut microbiota, immune responses, and IBS, has been highlighted. Among TLRs, TLR4 seems to be more involved in the development of IBS. TLR4 interacts with lipopolysaccharide (LPS) pattern, which is the most outer surface component of almost all Gram-negative bacteria (95, 96). It was demonstrated that stimulation of TLR4 by LPS can lead to motivation of the enteric nervous system and motility of the intestine (97). This finding was further investigated and supported by the comparison of the expression of TLRs in biopsy samples of patients with IBS and healthy controls in which the results implied a significant elevation of TLR4 in patients with IBS (98). Furthermore, Belmonte et al. (99) evidenced that the levels of TLR may be different based on the IBS subtypes. In this regard, they reported a significant upregulation of TLR2 and TLR4 in patients with IBS-M together with elevation of IL-8 and IL-1β (99). Recently, Jalanka et al. (100) analyzed the correlation of the gene expression of TLR4 and correlated receptors in patients with IBS and supported the probable role of a low inflammation due to bacteria in the intestine of patients with IBS. Therefore, it seems that a change in the gut microbiota composition may arrange a chronic inflammation and subsequent IBS.

## The Correlation Between *Blastocystis* sp. and the Gut Microbiota in Patients With IBS

Many studies demonstrated the effects of colonization of *Blastocystis* sp. on the gut microbiota, in both composition and richness. It was suggested that the presence of *Blastocystis* sp. reduced a Firmicutes*/*Bacteroidetes ratio (F/B ratio) in two cohort studies, FACSA and UNEME, which were performed in healthy people and individuals with metabolic disorders in Mexico (101). The mean proportions of *Faecalibacterium* spp. and Ruminococcaceae in the *Blastocystis* sp.-positive group, and *Enterococcus* spp. in the *Blastocystis* sp.-negative group were abundant in Korean populations (102). The high richness of *Faecalibacterium, Prevotella*, Ruminococcaceae UCG-002, Muribaculaceae, Rikenellaceae, Acidaminococcaceae, *Phascolarctobacterium*, and Ruminococcaceae UCG-005 in individuals who carry *Blastocystis* sp., and *E. hirae, E. faecalis, E. durans*, Enterococcaceae, Lactobacillales, and Bacilli in subjects who were negative for *Blastocystis* sp. was observed (102). Importantly, this study concluded that the presence of *Blastocystis* sp. is an indicator of healthy gut microbiota (102). Most recently, it was shown that *Blastocystis* sp. ST4 inhibited the growth of *B. vulgatus*, which suggests the protective role of *Blastocystis* sp. ST4 in the gut barrier integrity from damage due to the bacteria (103). Later, an altered gut microbial composition was documented in normal healthy mice and Rag1^−/−^ mice colonized by *Blastocystis* sp. ST4, mainly by an increased proportion of Clostridia vadinBB60 group and Lachnospiraceae NK4A136 group, respectively (104). These results confirmed the protective role of *Blastocystis* sp. ST4 in the modulation of the gut microbiota to reduce inflammation (104). *Blastocystis* sp. was reported to significantly increase alpha diversity in carriers. Accordingly, the presence of *Blastocystis* sp. was associated with enriched Firmicutes and Bacteroidetes, and the genera *Prevotella, Faecalibacterium, Flavonifractor, Clostridium, Succinivibrio*, and *Oscillibacter*, whereas Proteobacteria and the genera *Escherichia, Bacteroides, Klebsiella*, and *Pseudomonas* were enriched in *Blastocystis* sp.-negative group (105).

In contrast, a positive correlation between the presence of *Blastocystis* sp. and *C. difficile* was suggested in patients with IBD, which was also correlated with the number of defecation in these patients (10). Furthermore, Nourrisson et al. (106) investigated the gut microbiota in four classes of IBS and concluded that the presence of *Blastocystis* sp. was significantly correlated with an increase of *Lactobacilli* and a decrease of *Bifidobacterium* sp. and *F. prausnitzii* in male patients. Therefore, they suggested that *Blastocystis* sp. colonization may lead to a decrease in protective bacteria (106). To clear the role of *Blastocystis* sp., in shifting the gut conditions or gut microbiota composition in IBS, an experimental study was performed by Defaye et al. (107) in rats. Accordingly, they orally inoculated *Blastocystis* sp. ST4 in rats and evaluated the gut microbiota, inflammation, behavior, and short-chain fatty acid (SCFA). Interestingly, their findings showed that the presence of *Blastocystis* sp. was resulted in non-inflammatory colonic hypersensitivity with increased serine protease activity, anxiety- and depressive-like behaviors, the relative abundance of *Oscillospira* with a decrease in *Clostridium*, and lower levels of SCFAs (107), all of which highlight the role of *Blastocystis* sp. in the development of IBS and its symptoms, as well as microbiota shifting in patients with IBS (107).

**Figure 1:**
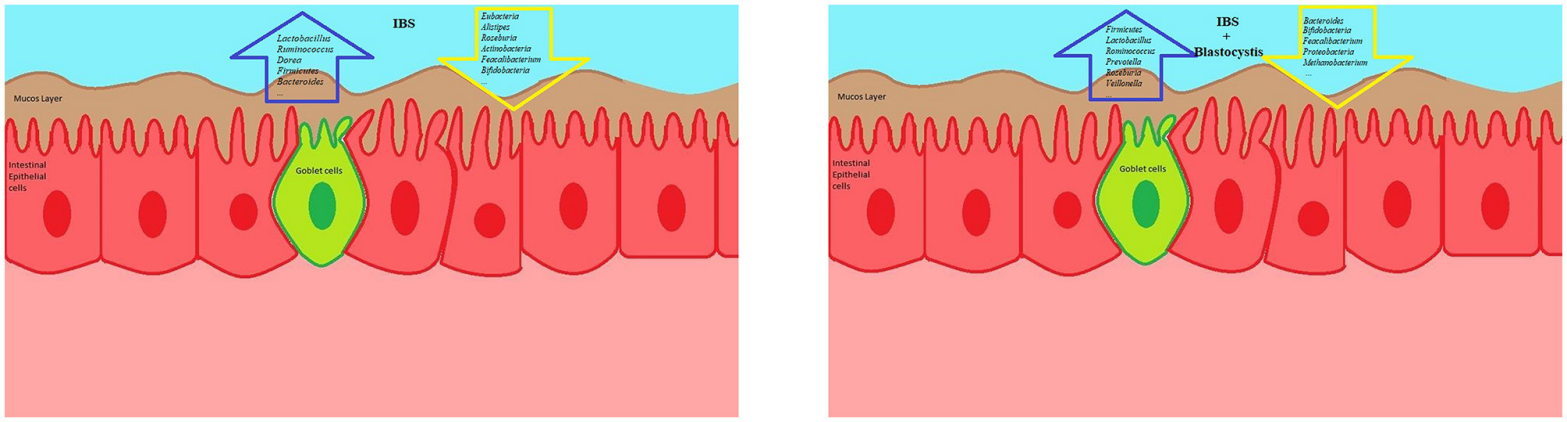
The schematic view presenting a correlation between *Blastocystis* sp. and certain microbiota signatures. The presence of *Blastocystis* sp. has not been linked with specific signature of gut microbiota. Although this protist seems to a healthy indicator for the gut microbiota, *Blastocystis* sp. can reduce a Firmicutes*/*Bacteroidetes ratio and protective bacteria. In addition, the low and high presence of *Blastocystis* sp. in IBD and IBS, respectively, might be related to the differences in the gut microbiota composition between IBD and IBS.

Recently, the eukaryome and prokaryome profiles of patients with IBS-C showed that the colonization of *Blastocystis* sp. not only induces changes in prokaryotic microbiota of the gut, particularly Tenericutes phylum and Ruminococcaceae family, but also this protist may disturb eukaryome population, particularly fungi, in patients with IBS (108). Importantly, gut mycobiome seems to play an important role in the development, presentation of symptoms, and response to treatment in patients with GI disorders (109–111). Nevertheless, the role of *Blastocystis* sp. in the gut microbiota changes is controversial, and there is evidence of a neutral role of this protist in the gut microbiota composition in patients with IBS (74). The controversial correlation between the gut microbiota composition and colonization of *Blastocystis* sp. (112) is thought to be related to antibiotic consumption (113). Remarkably, antibiotics can alter the gut microbiota composition and change the gut lumen from a favorable niche for *Blastocystis* sp. colonization toward an inimical condition for the protist (Figure 1 and Table 2).

**Table 2:** The gut microbiota changes in patients with IBS carrying *Blastocystis* sp.

**No**.	**Sample types**	**Age**	**Methods**	**Number of**	**Number of healthy**	**IBS patients**		***Blastocystis*** **-positive**		***Blastocystis*** **-positive**		**References**
				**patients**				**IBS patients**		**control subjects**		
						**Increase**	**Decrease**	**Increase**	**Decrease**	**Increase**	**Decrease**	
1	Stool	Adult	16S rRNA	40 (24 *Blastocystis* positive and 14 *Blastocystis* negative)	57 (42 *Blastocystis* positive and 15 *Blastocystis* negative)	Actinobacteria, Cyanobacteria, Elusimicrobia, Firmicutes, Fusobacteria, Proteobacteria, Methanobacteria, *Streptococcus*, *Clostridia*, Lachnospiraceae, Alphaproteobacteria, *Blautia*	Bacteroidetes, *Veillonella*, *Dialister*, *Catenibacter*, *Butyricimonas*, *Olsenella*	Bacteroidetes, Cyanobacteria, Firmicutes, Fusobacteria	Actinobacteria, Elusimicrobia, Proteobacteria,	–	–	(74)
2	Stool	Adult	qPCR 16S rRNA	56	56	Bacteroidetes, Firmicutes	–	–	Bifidobacteria, Faecalibacter, *Lactobacillus*	*Lactobacillus*	Bifidobacteria	(106)
3	Stool	Adult	16S rRNA qPCR	35	23	Proteobacteria Bacteroidetes Tenericutes	Firmicutes	Actinobacteria, Firmicutes	Bacteroidetes Proteobacteria	*Ruminococcus* *Bacteroidetes* *Tenericutes*	Firmicutes Actinobacteria Bacteroidetes Proteobacteria	(108)

## Concluding Remarks

*Blastocystis* sp. is a prevalent protist, which is reported from apparently healthy subjects, as well as individuals with a variety of GI disorders. Although the presence of *Blastocystis* sp. has not been linked with certain symptoms or disorders, this protist is reported in high prevalence rate in some diseases, such as IBS. IBS conditions seem to provide a favorable niche for colonization of *Blastocystis* sp. in the intestine. It is not clear how *Blastocystis* sp. communicates with the gut microbiota in healthy and disease conditions, particularly in patients with IBD and IBS. Some studies suggested that *Blastocystis* sp. is a healthy gut indicator, while the high prevalence of this protist in IBS proposes a correlation between improper gut conditions and *Blastocystis* sp. colonization. Nevertheless, recent studies have indicated a correlation between *Blastocystis* sp. and gut microbiota. It is not clear that either *Blastocystis* sp. may manipulate the gut microbiota composition or an altered gut microbiota in IBS may provide favorable conditions for colonization of *Blastocystis* sp.

Gut permeability is a key point of IBS development. *Blastocystis* sp. discharges a number of proteins, particularly a broad spectrum of proteases, which affect the gut permeability and tight junctions. Although limited data, the secretion levels and types of proteases and the effects of proteases on the human cells are supposed to be different in *Blastocystis* sp. strains. Therefore, the study of proteases, particularly cysteine protease, derived from different isolates and subtypes on the gut permeability and tight junction proteins provides interesting data. There is no documented study investigating the role of extracellular vesicles (EVs) discharged from either *Blastocystis* sp. or *Blastocystis* sp.-affected host cell on the gut permeability. Indeed, a cross-talk between *Blastocystis* sp. and gut microbiota *via* EVs may play a role in the successful colonization of the protist or the gut microbiota composition of the gut. EVs play an important role in cross-talk between microorganisms and the study of EVs (released from *Blastocystis* sp., gut microbiota, and/or host cells) would be interesting. However, researches on *Blastocystis* sp. is at the beginning stages and more studies are needed to be performed.

## Ethics Statement

This study was approved by the ethical standards (IR.IAU.SRB.REC.1400.241) released by Ethical Review Committees of Islamic Azad University Science and Research Branch, Tehran, Iran.

## Author Contributions

HM and ESM: conceived and designed. AO, HM, ESM, and AY: data gathering and literature review. AO and HM: writing the manuscript. AS: reviewing and editing the manuscript. All authors read and approved the final version of the manuscript.

## Conflict of Interest

The authors declare that the research was conducted in the absence of any commercial or financial relationships that could be construed as a potential conflict of interest.

## Publisher's Note

All claims expressed in this article are solely those of the authors and do not necessarily represent those of their affiliated organizations, or those of the publisher, the editors and the reviewers. Any product that may be evaluated in this article, or claim that may be made by its manufacturer, is not guaranteed or endorsed by the publisher.
